# Comparison of the ‘*Ca*. Liberibacter asiaticus’ Genome Adapted for an Intracellular Lifestyle with Other Members of the Rhizobiales

**DOI:** 10.1371/journal.pone.0023289

**Published:** 2011-08-18

**Authors:** John S. Hartung, Jonathan Shao, L. David Kuykendall

**Affiliations:** United States Department of Agriculture, Agricultural Research Service, Molecular Plant Pathology Laboratory, Beltsville, Maryland, United States of America; University of Osnabrueck, Germany

## Abstract

An intracellular plant pathogen ‘*Candidatus* Liberibacter asiaticus,’ a member of the *Rhizobiales*, is related to *Sinorhizobium meliloti*, *Bradyrhizobium japonicum*, nitrogen fixing endosymbionts, *Agrobacterium tumefaciens*, a plant pathogen, and *Bartonella henselae*, an intracellular mammalian pathogen. Whole chromosome comparisons identified at least 50 clusters of conserved orthologous genes found on the chromosomes of all five metabolically diverse species. The intracellular pathogens ‘*Ca*. Liberibacter asiaticus’ and *Bartonella henselae* have genomes drastically reduced in gene content and size as well as a relatively low content of guanine and cytosine. Codon and amino acid preferences that emphasize low guanosine and cytosine usage are globally employed in these genomes, including within regions of microsynteny and within signature sequences of orthologous proteins. The length of orthologous proteins is generally conserved, but not their isoelectric points, consistent with extensive amino acid substitutions to accommodate selection for low GC content. The ‘*Ca*. Liberibacter asiaticus’ genome apparently has all of the genes required for DNA replication present in *Sinorhizobium meliloti* except it has only two, rather than three RNaseH genes. The gene set required for DNA repair has only one rather than ten DNA ligases found in *Sinorhizobium meliloti*, and the DNA PolI of ‘*Ca*. Liberibacter asiaticus’ lacks domains needed for excision repair. Thus the ability of ‘*Ca*. Liberibacter asiaticus’ to repair mutations in its genome may be impaired. Both ‘*Ca*. Liberibacter asiaticus and *Bartonella henselae* lack enzymes needed for the metabolism of purines and pyrimidines, which must therefore be obtained from the host. The ‘*Ca*. Liberibacter asiaticus’ genome also has a greatly reduced set of sigma factors used to control transcription, and lacks sigma factors 24, 28 and 38. The ‘*Ca*. Liberibacter asiaticus’ genome has all of the hallmarks of a reduced genome of a pathogen adapted to an intracellular lifestyle.

## Introduction

Huanglongbing (HLB), arguably the most serious disease of citrus worldwide [1], [2], originated in South Asia [3], [4] and recently became epidemic in Brazil [5] and in Florida [6] homes of the largest orange juice industries in the world. ‘*Candidatus* Liberibacter asiaticus’ is consistently associated with HLB disease. Using electron microscopy, researchers observed the pathogen within sieve cells of phloem vessels of infected plant hosts [7], [8] or in the salivary glands of citrus psyllids, natural vectors of the pathogen [9]. The pathogen can also be readily detected by PCR-based tests, most based on the 16S rRNA gene [10], [11]. Placement of ‘*Ca*. Liberibacter asiaticus’ within the α-2 subdivision of the Proteobacteria class [12] was confirmed by sequence analysis of the 16S–23S RNA gene region [13]–[15]. In spite of many efforts [16]–[19], ‘*Ca*. Liberibacter asiaticus’ has not been artificially cultured. Thus the bacterium seems to have an obligate intracellular lifestyle in either plant or insect hosts.

In their landmark paper, Duan et al. provided a complete genomic sequence of ‘*Ca*. Liberibacter asiaticus’ strain Psy62 [20]. The sequence was obtained by deep sequencing of DNA obtained from an individual psyllid containing at least 8×10^8^ copies of the ‘*Ca*. Liberibacter asiaticus’ genome, based on q-PCR of its 16S rRNA gene. This genomic sequence data was confirmed by an independent group who also employed deep sequencing of the contents of phloem cells from infected trees obtained by laser micro dissection [21]. The genome is composed of a circular chromosome of 1.23 MB with no extra chromosomal elements. Analysis of the full genomic sequence enabled Duan et al. (2009) to place ‘*Ca*. Liberibacter asiaticus,’ within the Rhizobiaceae family of the order Rhizobiales [22]–[23] of the class α-Proteobacteria.

The intracellular bacterium ‘*Ca*. Liberibacter asiaticus’ and other intracellular members of the α-Proteobacteria, such as members of the genera *Rickettsia* and *Bartonella*, have reduced genome size when compared to free-living related bacteria [20], [24]. Intracellular bacteria also typically have genomes with remarkably low mol% G+C content [25], but there are also examples of free-living bacteria with larger genomes but equally low mol% G+C in their genomic sequence [26]. Forces that drive the reduction in genome size and the decrease in mol% G+C content remain obscure but are likely to result ultimately from intense selection for managing scarce resources and the lower energy costs associated with AT vs GC base pairs [27]. Intracellular organisms may have an enhanced rate of genomic evolution [28], and indeed ‘*Ca*. Liberibacter asiaticus’ occupies by far the longest branch in a phylogram of members of the Rhizobiales [20]. Other generalizations made about characteristics of reduced genomes include the reduction of ribosomal gene operons, the loss of some genes encoding enzymes needed for DNA repair, the loss of sigma factors needed to regulate transcription, and the loss or retention of different sets of genes that tend to be either dispensable or indispensable [29], [30].

When chromosomes are compared, phylogenetically-related bacteria typically share clusters of orthologous genes (COGs). We propose the term MOG (Microsyntenous Orthologous Genes) to avoid confusion with proteins described in the COG database meaning clusters of orthologous groups of proteins. We compared MOGs in ‘*Ca*. Liberibacter asiaticus’ with those in *Sinorhizobium meliloti*, *Bradyrhizobium japonicum* and *Agrobacterium tumefaciens*. These bacteria, capable of free-living, symbiotic nitrogen fixing, and pathogenic lifestyles are highly adaptable and thus have large genomes particularly when compared to the reduced genome of ‘*Ca*. Liberibacter asiaticus’. We therefore also compared the genome of ‘*Ca*. Liberibacter asiaticus’ to that of *Bartonella henselae*, a flea-vectored pathogen of cats and, opportunistically, of humans. Although *B. henselae* can be artificially cultured, it has complex nutritional requirements and thus it can be considered a semi-obligate intracellular pathogen of cats, reproducing in erythrocytes [31]. It possesses a severely reduced genome, not much larger than that of ‘*Ca*. Liberibacter asiaticus’ [31], [32].

We have studied these genomes in detail, within regions of conserved microsynteny between reduced and large genomes, and within signature regions of orthologous proteins. We have also performed global comparisons of gene sets whose members are often reduced in the genomes of intracellular pathogens. This analysis provides insight into the remarkable evolutionary pathway leading to a reduced genome in ‘*Ca*. Liberibacter asiaticus’ as compared to the larger genomes of other members of the *Rhizobiales* with free-living and plant-associated lifestyles.

## Results and Discussion

### Length and isoelectric point of orthologous proteins

**Figure 1:**
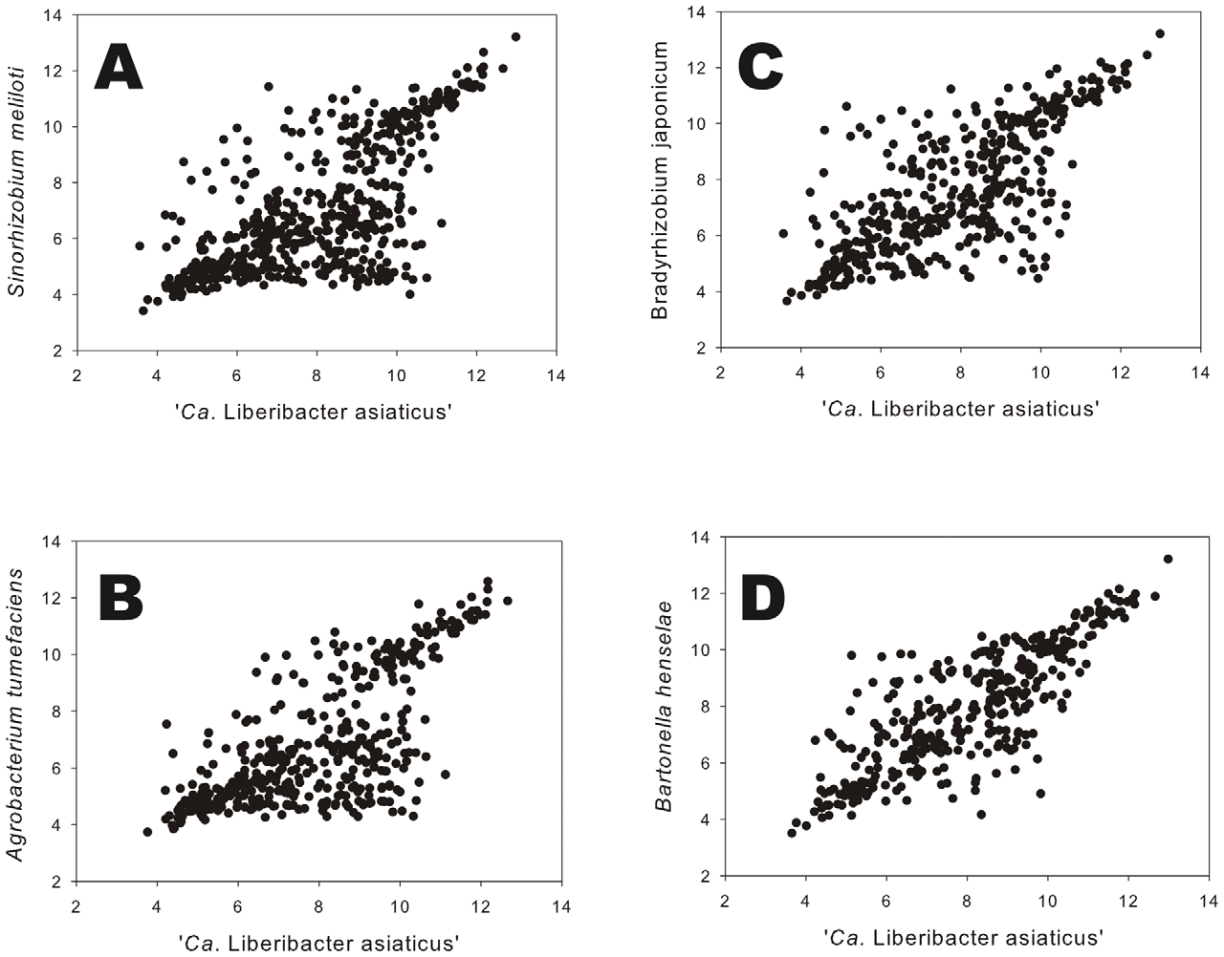
Isoelectric points of orthologous protein pairs. Orthologous proteins from ‘*Ca*. Liberibacter asiaticus’ and *Sinorhizobium meliloti* (A), *Agrobacterium tumefaciens* (B), *Bradyrhizobium japonicum* (C) and *Bartonella henselae* (D).

We identified orthologous protein pairs, and hence, conserved orthologous genes, based on BlastP e values of less than 10^−15^, a relatively conservative criterion. Manual curation of the resulting datasets increased the size of conserved clusters of genes by 10–15%. We compared orthologous protein pairs encoded by corresponding genes from microsyntenous orthologous groups. Isoelectric points (pIs) and the number of amino acids predicted for orthologous protein pairs were plotted for ‘*Ca*. Liberibacter asiaticus’ vs *S. meliloti*, *B. japonicum*, *A. tumefaciens*, and *B. henselae*. Although there seems to be a general tendency for the pIs of orthologous protein pairs to correlate, the correlation was weak and substantial variation was observed (Fig. 1). Nonetheless, we observed generally higher pI's for proteins of ‘*Ca*. Liberibacter asiaticus’ as compared to their orthologs in the free living Rhizobiales. For example, the mean pI for 552 orthologous proteins for ‘*Ca*. Liberibacter asiaticus vs *S. meliloti* was 7.90 vs 6.91. Similar results were found for 426 orthologous proteins from ‘*Ca*. Liberibacter asiaticus’ vs *A. tumefaciens*. However, when 362 orthologous proteins from the intracellular pathogens were compared, the pIs were nearly the same, 8.02 for ‘*Ca*. Liberibacter asiaticus’ and 7.90 for *B. henselae*.

**Figure 2:**
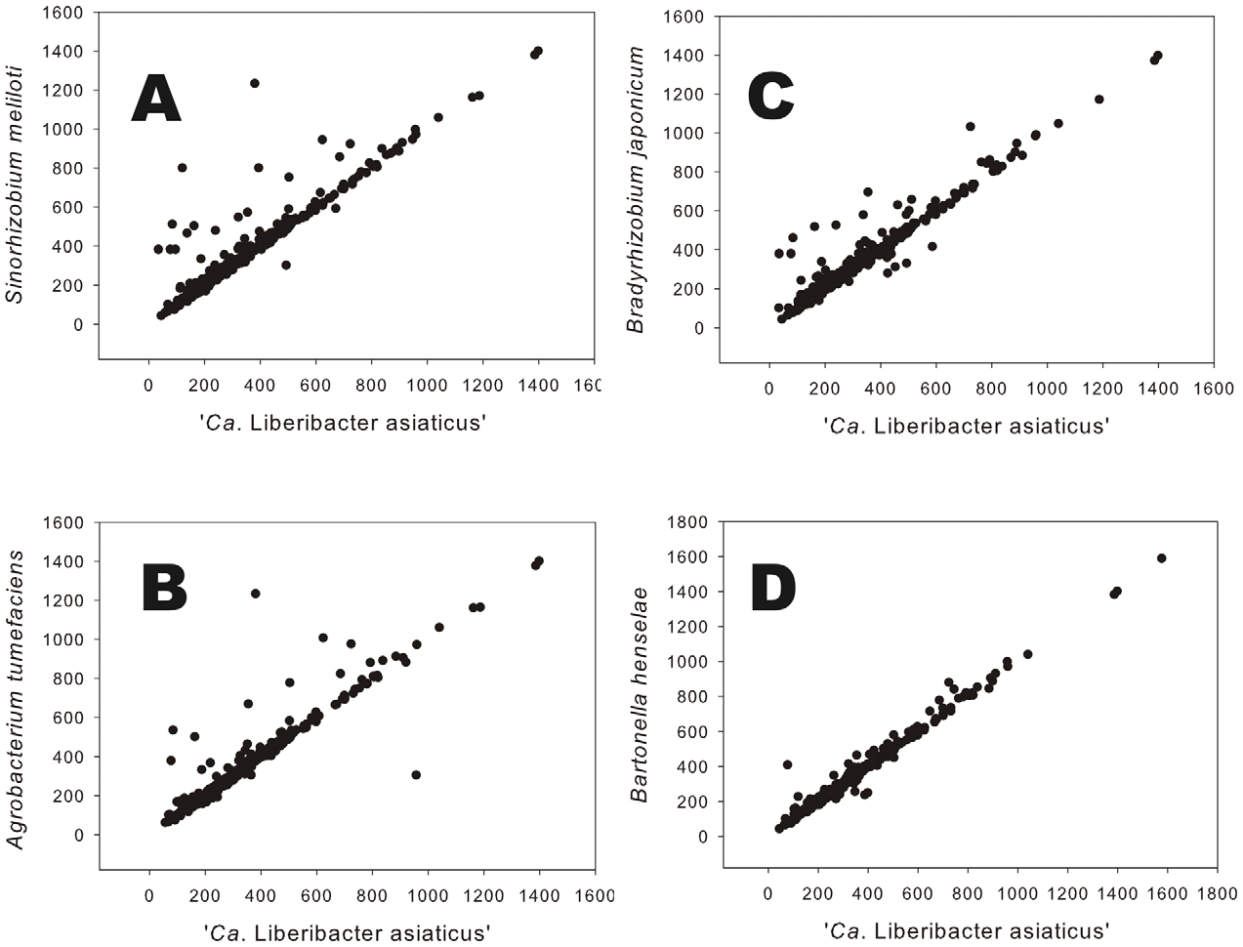
Number of amino acids in orthologous protein pairs. Orthologous proteins from ‘*Ca*. Liberibacter asiaticus’ and *Sinorhizobium meliloti* (A), *Agrobacterium tumefaciens* (B), *Bradyrhizobium japonicum* (C) and *Bartonella henselae* (D).

We also plotted the number of amino acids in orthologous protein pairs in microsyntenous orthologous groups and found that the correlation in length of orthologous protein pairs was much stronger (Fig. 2). Amino acid plots of orthologous protein pairs shared between *S. meliloti* and ‘*Ca*. Liberibacter asiaticus’ and orthologous protein pairs shared between *B. japonicum* and ‘*Ca*. Liberibacter asiaticus’ revealed several proteins pairs well separated from the diagonal. For the most part, these fell above the diagonal, indicating that the orthologous protein in ‘*Ca*. Liberibacter asiaticus’ was shorter in length than the corresponding protein in *S. meliloti* or *B. japonicum*. In contrast, the correlation between the lengths of each member of orthologous protein pairs was very high for ‘*Ca*. Liberibacter asiaticus’ and *B. henselae*, the two bacteria with similar intracellular parasitic lifestyles and reduced genomes (Fig. 2).

The relatively high mean isoelectric points of proteins from ‘*Ca*. Liberibacter asiaticus’ as compared to their orthologs from free living bacteria, is similar to other intracellular pathogens [33]. The mean of the isoelectric points of proteins from ‘*Ca*. Liberibacter asiaticus’ were not different from that of orthologs in *B. henselae*, consistent with both bacteria being intracellular pathogens with reduced genomes. The isoelectric points (pIs) of orthologous protein pairs are not under stringent selection since conservative substitutions of amino acids can retain function while changing the pI [34], [35].

When the pIs of orthologous protein pairs from the closely related *S. meliloti* and *A. tumefaciens* were individually compared graphically [34], a great deal of dispersion away from the diagonal was observed and subsets of protein pairs were considered to be differentially grouped away from the diagonal [34]. Such grouping of protein pairs away from the diagonal was not observed in any of the graphical comparisons made here between ‘*Ca*. Liberibacter asiaticus’ and the other members of the Rhizobiales; instead the dispersion from the diagonal was continuous but was noticeably less when *B. henselae* and ‘*Ca*. Liberibacter asiaticus’ were compared. The lack of correlation of pIs between orthologous proteins of ‘*Ca*. Liberibacter asiaticus’ when compared to those of other members of the Rhizobiales is consistent with phylogenetic distance [20] and the extreme shift in codon usage between ‘*Ca*. Liberibacter asiaticus’ and free-living members of the Rhizobiales. However, the relative lengths of orthologous proteins was well conserved despite a consistent trend toward overall reduction in the obligate intracellular microorganism, extending data from other systems [34], [35] to intracellular pathogens with extremely reduced genomes.

### GC content in microsyntenous orthologous groups of paired genomes

**Figure 3:**
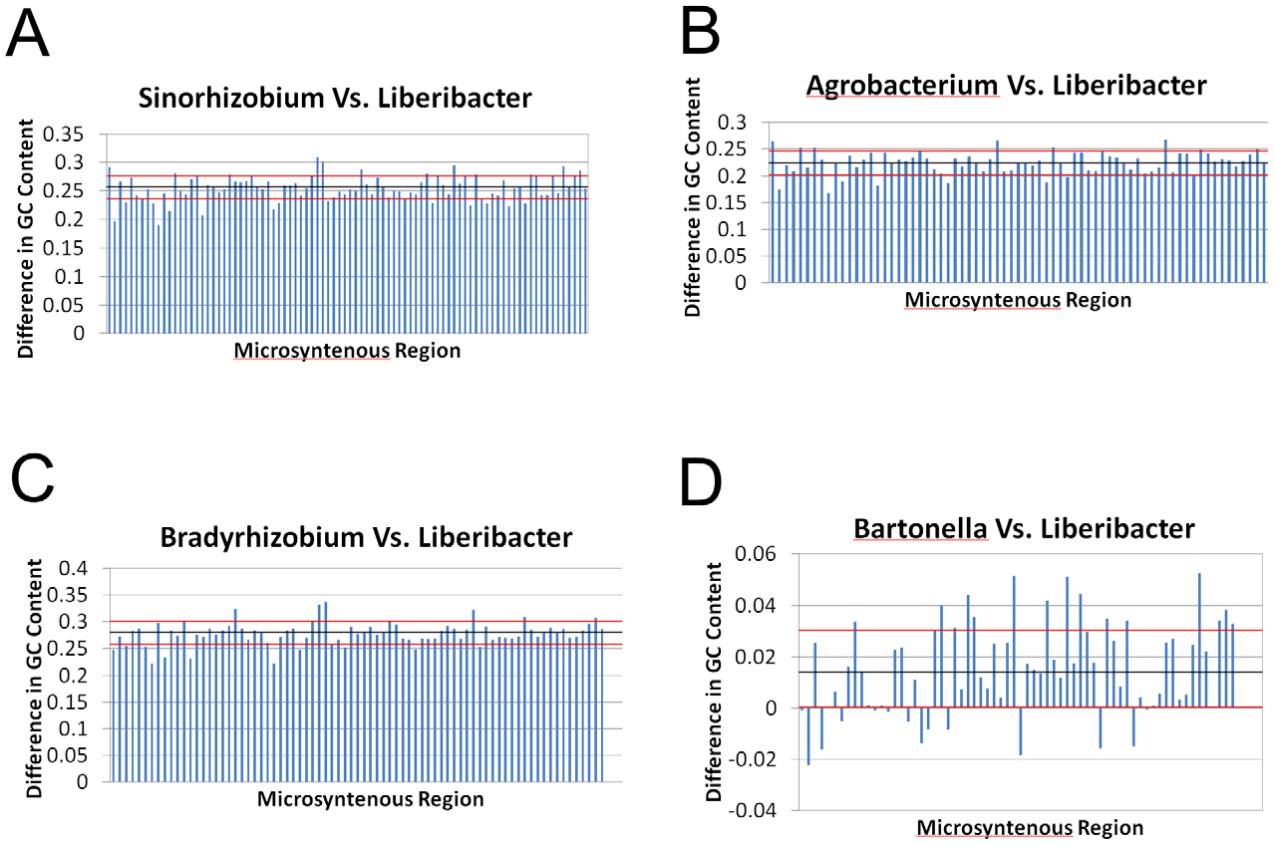
Differences in GC content in microsyntenous orthologous groups. The GC content of microsyntenous orthologous groups of ‘*Ca*. Liberibacter asiaticus’ compared with other members of the Rhizobiales. The dark bar and red lines denote the mean and one standard deviation of the mean.

Ninety-one microsyntenous orthologous groups, (MOGs), were identified when the genomes of ‘*Ca*. Liberibacter asiaticus’ and *S. meliloti* were compared. The mol% GC content of the microsyntenous MOG regions in *S. meliloti* was 25.5±2.1 percent greater than the corresponding regions of ‘*Ca*. Liberibacter asiaticus (Fig. 3). The MOGs of *B. japonicum* had 27.8±2.1 mol% higher GC content than their orthologous MOGs in ‘*Ca*. Liberibacter asiaticus’ (Fig. 3). In contrast, when the GC content of 66 MOGs conserved between ‘*Ca*. Liberibacter asiaticus and *B. henselae* were compared, the differences in mol% GC content were small, only 1.5±0.2 percentage points. These data are consistent with the overall GC content of the respective genomes, and the fact the *B. henselae*, like ‘*Ca*. Liberibacter asiaticus’ has a highly reduced genome.

To determine if the preference for AT-rich codons held in the functionally and structurally conserved motifs of the encoded proteins, five arbitrarily selected sets of MOGs from ‘*Ca*. Liberibacter asiaticus’ and *S. meliloti* were chosen for comparative study. The percentage of GC base pairs in the genomic sequences encoding the functional domains of the proteins was consistent with the overall GC usage for each genome, with ‘*Ca*. Liberibacter asiaticus’ and *S. meliloti* having GC content of 35–40% and 60–65% respectively in the functional domains of these orthologous proteins.

Orthologous proteins have been retained in ‘*Ca*. Liberibacter asiaticus’ without conservation of the coding sequences, but with a low GC bias for the genome as a whole. This low G+C codon bias is often observed in intracellular bacteria, and mathematical analysis has shown that such synonymous codon usage bias is dominated by selection at the third codon position [36] . The evolutionary driver of the low GC bias may very likely be related to the higher energy cost to synthesize a GC base pair relative to an AT base pair in the genome [27], as a part of a larger competition for limited metabolic resources from the host. In addition to the energy saved in the synthesis of AT vs GC base pairs, the thermodynamic stability of AT rich sequences is lower than that of GC rich sequences. DNA helicase consumes ATP in order to separate DNA strands [37]. Therefore we hypothesize that replication and transcription of an AT rich genome will be less energetically expensive than that of a GC rich genome, based on a lower ATP cost for strand separation. This contributes to the evolution of AT rich genomes in intracellular parasites where competition for resources with the host is intense.

### Codon preference and amino acid usage in proteins of ‘*Ca*. Liberibacter asiaticus’ vs other members of the Rhizobiales

Amino acid codon usage by ‘*Ca*. Liberibacter asiaticus’ was distinctly different from that of *A. tumefaciens*, *B. japonicum* and *S. meliloti*, consistent with an overall drastic reduction in GC content of the genome (Table S1). For example, across the entire ORFeome, ‘*Ca*. Liberibacter asiaticus’ the codon ‘CA**A**’ for glutamine was used 73% of the time while *A. tumefaciens*, *B. japonicum* and *S. meliloti* used the codon ‘CA**G**’ for glutamine more than 80% of the time. ‘*Ca.* Liberibacter asiaticus’ ‘preferred’ the codon ‘TG**T**’ for cysteine (75%) while *A. tumefaciens*, *B. japonicum* and *S. meliloti* used the codon ‘TG**C**’ for cysteine more than 80% of the time. Similarly, codons ‘TT**A**’, ‘CT**A**’, or ‘CT**T**’ were used by ‘*Ca*. Liberibacter asiaticus’ to encode leucine 65% of the time but were used only about 15% of the time by *A. tumefaciens*, *B. japonicum* and *S. meliloti*. These bacteria instead used codons ‘CT**G**,’ ‘TT**G**’ or ‘CT**C**’ about 88% of the time. Reduced usage of G and C nucleotides in triplet codons determining a given amino acid incorporated into protein is consistent throughout the ‘*Ca*. Liberibacter asiaticus’ genome. *B. henselae* shared the strong preference for AT-rich amino acid codons in the ORFeome with ‘*Ca*. Liberibacter asiaticus’ (Table S1). The preference for A or T codons at the third position of amino acid codons in intracellular bacteria has been observed previously [36]. This is consistent with rapid evolution of genes in bacterial endosymbionts relative to those of free living bacteria, a phenomenon reflected in amino acid sequences of proteins [28].

Genome-wide comparisons also showed that usage of the amino acids proline, arginine, valine, glycine and alanine was lower in *B. henselae* and ‘*Ca*. Liberibacter asiaticus’ than in the free-living Rhizobiales. Usage of the amino acids phenylalanine, serine, cysteine, glutamine, isoleucine, asparagine, lysine was higher in *B. henselae* and ‘*Ca*. Liberibacter asiaticus’ as compared with that of the free-living Rhizobiales (Table S2). The tRNA synthetases corresponding to these amino acids are present in the proteome. Thus, the differences in usage are not likely to be due to an inability of ‘*Ca*. Liberibacter asiaticus’ to synthesize amino acids *de novo* due to loss of metabolic capability with the reduced genome. Instead, we observe that there is a strong correlation between the AT content of the codons for amino acids and the frequency of occurrence of the amino acids in the ‘*Ca*. Liberibacter asiaticus’ and *B. henselae* genomes (Table S2).

### Purine and pyrimidine metabolism

‘*Ca*. Liberibacter asiaticus’ is markedly deficient in its ability to carry out purine and pyrimidine metabolism as compared to *S. meliloti* (Figures S1 and S2). ‘*Ca*. Liberibacter asiaticus’ retains only 47 genes annotated for purine metabolism compared with the 95 genes annotated for purine metabolism in the genome of *S. meliloti*. Although it can produce the nucleotides GTP and ATP from their respective nucleoside diphosphates, ‘*Ca*. Liberibacter asiaticus’ does not appear able to synthesize guanine, xanthine, hypoxanthine or adenine. Likewise, ‘*Ca*. Liberibacter asiaticus’ retains only 38 genes for pyrimidine metabolism compared with the 60 genes for pyrimidine metabolism in the genome of *S. meliloti*. Although ‘*Ca*. Liberibacter asiaticus’ can produce UTP and CTP from their respective nucleoside diphosphates it does not appear to be able to produce uracil, cytosine or thymine.

‘*Ca*. Liberibacter asiaticus,’ likely has lost the ability to synthesize and metabolize both purines and pyrimidines, and these may have to be obtained from the host as di- or tri-nucleosides. The loss of these pathways would be consistent with a general bias towards deletion of genes in the process leading to reduced genomes if the gene products become redundant in the intracellular environment [38]. The incorporation of uracil into DNA by either replication error or cytosine to uracil deamination has been proposed as a driver towards the low GC/high AT genome, particularly if DNA repair if ineffective [33], as is likely to be the case here. In this context it is notable that E.C. 3.5.4.1, used to interconvert cytosine and uracil, is missing in ‘*Ca*. Liberibacter asiaticus’. This defect has been noted previously in *Ureaplasma urealyticum*, another intracellular bacterium [25]. A caveat is necessary here. The lack of annotated orthologous proteins to perform a particular task in ‘*Ca*. Liberibacter asiaticus’ does not mean that the organism cannot perform that task. Several examples have been described in *Mycoplasma* spp. where relaxed substrate specificity for enzymes compensated for loss of function [39]. Examples of enzymes with relaxed specificities included kinases [40] and malate/lactate dehydrogenase [41]. Still, the apparent loss of metabolic capability is striking.

### DNA replication and repair

Atypical and reduced DNA replication complements are typically observed in reduced genomes [30]. Two critical components of the DNA polymerase III complex, the alpha and gamma/tau subunits, were annotated as pseudogenes in the original description of the ‘*Ca*. Liberibacter asiaticus’ genome [20]. We have conducted a complete bioinformatic analysis of the DNA replication complex in ‘*Ca*. Liberibacter asiaticus’ (Table 1). The DNA polymerase III alpha subunit (COG0587) is located between genes CLIBASIA_03630 and CLIBASIA_03645 in the Liberibacter genome and thus occurs between positions (797277–801416). The protein length is 1000+ amino acids long and is characteristic of other DNA polymerase III alpha subunits. SMART analysis reveals a pFAM DNA_pol3_alpha domain. There is an open reading frame using a triplet codon encoding leucine as the starting amino acid (797696–800797) with a strong blast hit to *Rhizobium etli* DNA polymerase III alpha subunit (YP_001977851.1, E-value 0.0). This protein is thus presumed to be functional. DNA polymerase III subunit gamma/tau (COG2812) is located between genes CLIBASIA_03275 and CLIBASIA_03260 in the Liberibacter genome. The protein is 370 amino acids long, shorter than other DNA Pol III gamma/tau subunits. However Smart analysis shows an ATPase domain and there is an open reading frame from nucleotide position 499568 to 500677. This protein has an e-value of 9.0E-151 when compared to its ortholog in *S. meliloti*, and the domains of the ‘*Ca*. Liberibacter asiaticus’ and *S. meliloti* proteins show similar e-values when compared to the pFAM models (Table 1). The rest of the DNA replication complex is also present with low e-values values to both orthologs in *S. meliloti* and functional domains in pFAM. The major difference between *S. meliloti* and ‘*Ca*. Liberibacter asiaticus’ in the DNA replication complex is that *S. meliloti* encodes three RNase H orthologs but ‘*Ca*. Liberibacter asiaticus’ encodes only two orthologs of RNase H (Table 1).

**Table 1:** Proteins involved in the DNA Polymerase III replication complex in ‘*Ca*. Liberibacter asiaticus’ and *Sinorhizobium meliloti*.

ANNOTATION	Protein in	Protein in	E value to	L. asiaticus	L. asiaticus	*S. meliloti*	*S. meliloti*
	L. asiaticus	S.meliloti	L. asiaticus to	pFAM	E-value to	pFAM	E-value to
			*S. meliloti*	Domains	pFAM model	Domains	pFAM model
Epsilon	ACT57291	CAC41392	6.00E-67	EXO III	5.91E-39	EXO III	9.44E-47
Alpha	N/A^a^	CAC45868	0	DNA Pol 3 α	3.40E-267	DNA Pol 3 α	8.20E-302
				Pol IIIAc	1.15E-18	Pol IIIAc	4.60E-18
Gamma/Tau	N/A^b^	CAC41667	9.00E-151	AAA	1.54E-08	AAA	3.52E-12
Delta'	ACT56690	CAC46182	2.00E-78	None^c^	None	None	None
Beta	ACT56927	CAC41772	2.00E-99	Pol 3Bc	8.67E-87	Pol3Bc	1.76E-119
Chi	ACT56864	CAC45735	5.00E-21	DNA Pol3 chi	4.60E-08	DNA Pol3 chi	2.50E-43
Delta	ACT57277	CAC47912	4.00E-45	DNA Pol3 δ	3.60E-04	DNA Pol3 δ	3.30E-05
DNAB	ACT56805	CAC45710	0	DNAB	9.30E-35	DNAB	1.40E-32
DNAG	ACT57307	CAC46897	3.00E-164	ZnF CHCC	3.34E-23	ZnF CHCC	2.76E-24
				Toprim N	1.90E-57	Toprim N	1.20E-61
SSB	ACT56656	CAC46137	3.00E-46	SSB	3.60E-32	SSB	1.90E-31
RNaseH	ACT57269	CAC45486	5.00E-49	RNase H	3.00E-47	RNase H	8.40E-58
	ACT56750	CAC45411	2.00E-53	RNase HII	4.20E-63	RNase HII	2.60E-62
	------------	CAC45163	-----------	----------	----------	RNase H	5.50E-40
DNA Pol I		CAC41562	0	53EXOc	7.69E-111	53EXOc	2.40E-121
				HhH2	4.15E-11	HhH2	1.10E-15
				POLAc	2.19E-106	POLAc	2.91E-120
Ligase	ACT57645	CAC46743	0	LIGANc	9.68E-172	LIGANc	1.30E-227

a Protein list compiles from the Kyoto Encyclopedia of Genomes and Genes.

b These proteins were originally annotated as pseudogenes and so a protein designation is not available.

c This protein does not match any pFAM domain.

The process leading to genome reduction such as has occurred in ‘*Ca*. Liberibacter asiaticus’ is thought to be facilitated by deficient DNA repair capability [29], [33]. We therefore searched the ‘*Ca*. Liberibacter asiaticus’ genome for the suite of enzymes active in DNA repair by comparison with the suite of DNA repair enzymes present in *S. meliloti*. Most such enzymes present in *S. meliloti* were also present in ‘*Ca*. Liberibacter asiaticus’, and are likely to be in functional condition based on the e-values between orthologs in *S. meliloti* and ‘*Ca*. Liberibacter asiaticus. We also found similar matches of functional domains of the proteins from these two bacteria to the pFAM database (Table S3). The noteworthy exceptions were for DNA PolI and DNA ligase. In the case of DNA PolI, the ortholog in ‘*Ca*. Liberibacter asiaticus’ was lacking domains 53EXOc and 35EXOc, which are expected to be particularly important in DNA excision repair. Both bacteria had three DNA ligase enzymes annotated on their chromosome, but two of these proteins were apparently not functional in ‘*Ca*. Liberibacter asiaticus’, as the e-values to *S. meliloti* orthologs were poor and there was no match at all to the model proteins in pFAM. It is important to note that we found an additional eight ligases encoded on pSymA and pSymB of *S. meliloti*, and 2 ligases encoded on the *A. tumefaciens* chromosome as well as five more ligases encoded on the linear chromosome and the At and Ti plasmids. *B. japonicum* has 10 ligases encoded on its chromosome (Table S4).

The accelerated rate of evolution observed within obligate intracellular bacteria [20], [28] may be facilitated by inefficient repair of mutations caused by loss of genes encoding DNA repair enzymes. ‘*Ca*. Liberibacter asiaticus’ is likely to be deficient in DNA repair as compared to *S. meliloti*. DNA ligase participates in all three DNA repair pathways, and so having only one ligase rather than eleven ligases as found in *S. meliloti* or 10 ligases as found in *B. japonicum* very likely limits the efficiency of DNA repair processes. Likewise, the lack of the EXO53 and EXO35 domains in the DNA PolI enzyme of ‘*Ca*. Liberibacter asiaticus’ is very likely to impede the excision repair process, and is also an example of an enzyme evolving relaxed substrate specificity concurrent with genome reduction [39], [40].

### Sigma factors

The genome of ‘Ca. Liberibacter asiaticus’ encodes only three sigma subunit factors, which enable differential transcription of genes by RNA polymerase (Table 2). These include one sigma 70 for genes expressed during exponential growth, one sigma 54 for genes involved in nitrogen metabolism and one sigma 32, involved in the heat shock or other stress responses. In contrast, the *S. meliloti* encodes 11 sigma factors, including three sigma 54 subunits and four sigma 24 subunits which enable expression of factors needed for the heat shock response as well as for factors exported from the cell. *S. meliloti* also has a special sigma factor, FecI for the expression of genes needed for the uptake of iron.

**Table 2:** Sigma factors present in the genomes of *Sinorhizobium meliloti* and ‘*Ca*. Liberibacter asiaticus.’

Sinorhizobium	Liberibacter	Identification	E. coli	Liberibacter vs	Function
Protein	Protein		gene	Sinorhizobium	
				E value	
CAC46893	ACT56762	sigma 70	rpoD	0	Exponential growth
CAC47374	ACT57389	sigma 54	rpoN	9.00 E-80	Nitrogen metabolism
CAC41817	N/A	sigma 54	rpoN	___________	Nitrogen metabolism
CAC41818	N/A	sigma 54	rpoN	___________	Nitrogen metabolism
N/A	N/A	sigma 38	rpoS	___________	stationary phase
CAC47305	ACT57084	sigma 32	rpoH	6.00 E-124	heat shock
CAC47835	N/A	sigma 32	rpoH	___________	heat shock
N/A	N/A	sigma 28	rpoF	___________	flagella
CAC46681	N/A	sigma 24	rpoE	___________	heat shock and export
CAC47413	N/A	sigma 24	rpoE	___________	heat shock and export
CAC46954	N/A	sigma 24	rpoE	___________	heat shock and export
CAC47014	N/A	sigma 24	rpoE	___________	heat shock and export
CAC46610	N/A	FecI	FecI	___________	Iron uptake

It is notable that ‘*Ca*. Liberibacter asiaticus’ has no recognizable sigma factors 24 or 38, which in *E. coli* are used for the expression proteins intended for export or for expression in the stationary phase of growth, respectively [42]. Neither *S. meliloti* nor ‘*Ca*. Liberibacter asiaticus’ encodes a sigma factor 28 which is required for expression of flagellar genes in *E. coli* [42]. *S. meliloti* produces flagellae so one of the other sigma subunits is apparently responsible for this activity in *S. meliloti*. Flagellae have not been observed in ‘*Ca*. Liberibacter asiaticus’, though an extensive set of flagellar genes is present [20].

The early workers who created the proposed genus ‘*Candidatus* Liberibacter’ placed it within the α-subdivision of the Proteobacteria based on sequence analysis of the 16S rRNA gene [12], [14]. Duan et al. [20] created a phylogenetic tree of the α-Proteobacteria based on concatenated sequence alignments of 94 proteins. Their overall alignment of the α-Proteobacteria was as previously demonstrated [43], with the addition of ‘*Ca*. Liberibacter asiaticus’ firmly within the Rhizobiales as an “early branching member” of the Rhizobiaceae. We took advantage of the relationship of ‘*Ca*. Liberibacter asiaticus’ with other members of the Rhizobiales to design the experiments described herein. Although the large regions of synteny previously observed in other similar comparisons [34] has been largely obscured by the process of genomic reduction, we have identified numerous microsyntenous orthologous groups of proteins that are conserved across all five species studied (Kuykendall et al., in preparation). The existence of so many MOGs in these inter specific genomic comparisons reflects the underlying evolutionary relationships among these species.

The ‘*Ca*. Liberibacter asiaticus’ genome is typical of reduced genomes adapted for the niche of intracellular parasitism [24], [29]–[30], [39] and in this regard is very similar to the genome of *Bartonella henselae* [32]. Orthologous protein pairs were identified and plotted against each other based on size and isoelectric points. The lengths of proteins in orthologous pairs, as well as the isoelectric points of the orthologous proteins, were very well correlated between the two intracellular pathogens. This is consistent with the greatly reduced genome sizes of ‘*Ca*. Liberibacter asiaticus’ and *Bartonella henselae* as compared with the other species. Both bacteria use a specialized genetic code and the amino acid composition in proteins is altered to minimize the occurrence of guanine and cytosine in their genomes, presumably because guanine and cytosine are more energetically expensive to make than are adenine and thymidine [27]. We further suggest that because the unwinding of the double helix to allow replication or transcription is done at the expense of ATP [37] , a genome rich in AT base pairs should be relatively less costly to replicate because of the lower thermodynamic stability of AT as compared with GC base pairs.

The GC content of the chromosomal DNA of ‘*Ca*. Liberibacter asiaticus’ and *B. henselae* is 26–27 mol% less than that of the other members of the Rhizobiales, consistent with other obligate intracellular pathogens with reduced genomes [24]. We show that the low GC content is achieved by a global and systematic reordering of the transcription process to favor AT vs GC rich codons for amino acids, such as phenylalanine and proline, when such a choice is available in the universal code. This AT preference extends to the preferred usage of TAA as the ‘stop’ codon over TGA in the other Rhizobiales. In any case GC content ought not to be a taxonomic criterion, as members of the well accepted order *Rhizobiales* within the α-Proteobacteria are shown here to have widely divergent GC content.

Although the DNA replication machinery appears to be intact in ‘*Ca*. Liberibacter asiaticus’, the relative efficiencies of orthologs from ‘*Ca*. Liberibacter asiaticus’ and *S. meliloti* have not been compared, so the relative efficiency of DNA replication between the two bacteria can not be accurately estimated.

The complete and uniform revision of the genome to favor AT rich codons over GC rich codons requires a massive accumulation of mutations. The capacity of ‘Ca. Liberibacter asiaticus’ to repair DNA appears to be limited, which would likely contribute to the rapid evolution of the genomic sequence observed [20], [28]. DNA PolI of ‘*Ca*. Liberibacter asiaticus’ lacks both of the exonuclease domains used for excision repair, and ‘*Ca*. Liberibacter asiaticus’ has only one ligase enzyme used more generally in DNA repair. The lack of capacity for DNA repair may represent a potential vulnerability for ‘*Ca*. Liberibacter asiaticus.

Likewise the lack of endogenous metabolic capability of ‘*Ca*. Liberibacter asiaticus’, as shown by its apparent inability to synthesize purines or pyrimidines may also represent vulnerabilities to ‘*Ca*. Liberibacter asiaticus’. The repertoire of sigma subunit factors is also greatly reduced in ‘*Ca*. Liberibacter asiaticus’ as compared to *S. meliloti*. Loss of sigma subunits is common in reduced genomes of bacteria adapted to an intracellular lifestyle [30]. Because of the relative richness of sigma factor subunits encoded by the *S. meliloti* genome, gene expression in *S. meliloti* is apparently under much more nuanced control than is the case with ‘*Ca*. Liberibacter asiaticus’, which is adapted to a stable host cell environment requiring few sigma factors. The apparent lack of nuanced control of genetic expression may present another weakness for ‘*Ca*. Liberibacter asiaticus’. We have noted a number of reductions in the ‘*Ca*. Liberibacter asiaticus’ genome that lead to a markedly reduced metabolic capability as well as reduced capabilities for DNA repair and fine control of genetic expression. Nonetheless, this reduced genome provides ‘*Ca*. Liberibacter asiaticus’ with the means to successfully infect and multiply to high levels in several plant species [7], [44] as well as within the insect vector *Diaphorina citri* [20] with whom it is also highly adapted. Thus the reduced genome is very subtly adapted to the intracellular environment, which is perhaps surprisingly similar between plant and insect hosts.

## Materials and Methods

Orthologous protein pairs were identified by P-BLAST alignments from genomic sequence data using a cut off e-value of less than 10^−15^. Microsyntenous regions that are conserved between the ‘*Ca*. Liberibacter asiaticus’ chromosome and those of four other members of the Rhizobiales were identified by using criteria that included (1) at least three orthologous genes in succession, (2) in the same order and (3) with predicted protein products that shared Blast alignment e values of less than 10^−15^ (Kuykendall et al., in preparation).

### Identification of orthologous proteins in microsyntenous orthologous groups

To identify orthologous genes and characterize them in some detail, amino acid sequences (‘*Ca*. Liberibacter asiaticus’ strain psy62, CP001677; *Bartonella henselae* strain Houston-1, BX897699; *Sinorhizobium meliloti* strain 1021, AL591688; *Bradyrhizobium japonicum* strain USDA 110, BA000040 and *Agrobacterium tumefaciens* strain C58, AE007869) were downloaded from NCBI and used with the author's original annotation. ‘*Ca*. Liberibacter asiaticus’ strain psy62 does not have plasmid DNA, and extra chromosomal DNA sequences were not included in this study except as noted. Using default BLAST parameters, each amino acid sequence from a chromosome of this group of strains was blasted against the amino acid sequences of the other chromosomes of this group of phylogenetically related strains. Perl scripts and Excel spreadsheets were created to easily identify hits between genomes with low, negative e-values.

### Calculation of GC content and amino acid and codon usage

A perl script was written to calculate the GC content of each gene in each syntenic block. The GC composition of syntenic blocks were compared between the genome of ‘*Ca*. Liberibacter asiaticus’ and the other members of the *Rhizobiales* and summarized graphically. Another perl script was written calculating codon usage for each gene in all 5 genomes. The percentage of occurrence of each amino acid and each amino acid codon were also calculated for each genome. The amino acid sequences of signature domains of proteins in selected syntenic blocks were determined using the SMART program (http://smart.embl-heidelberg.de/). The nucleotide sequence encoding these domains was extracted and the codon usage and GC content was calculated.

We also calculated the isoelectric points and the number of amino acids of orthologous proteins encoded in microsyntenous blocks of each genome. Isoelectric points of each protein in each syntenic block were calculated with the mw iep program (http://gchelpdesk.ualberta.ca/). The isoelectric points and number of amino acids in orthologous protein pairs in microsyntenous regions identified in the ‘*Ca*. Liberibacter asiaticus’ proteome as compared to the other members of the Rhizobiales were plotted against each other (Sigma Plot 11, Systat, San Jose, CA).

The functional capabilities of proteins involved in DNA repair and DNA replication pathways were estimated by comparing the proteins from ‘*Ca*. Liberibacter asiaticus to orthologous proteins from *S. meliloti*. Comparisons included e-values of the proteins to each other as well as e values of each protein to the pFAM models for each protein. Sigma factors were identified by reciprocal searches of the genomes of *S. meliloti* and ‘*Ca*. Liberibacter asiaticus’. The purine and pyrimidine metabolic networks were taken from the Kyoto Encyclopedia of Genomes and Genes. Orthologous genes in *S. meliloti* and ‘*Ca*. Liberibacter asiaticus’ were mapped on to these networks.

## Supporting Information

Figure S1 **Purine metabolism in** ***Sinorhizobium meliloti*** **and ‘** ***Ca*** **. Liberibacter asiaticus’.** Enzymes annotated as present in both ‘*Ca*. Liberibacter asiaticus’ and *S. meliloti* are colored green. Enzymes annotated as present in *S. meliloti* but not in ‘*Ca*. Liberibacter asiaticus are colored orange. The metabolic pathway is from the Kyoto Encyclopedia of Genomes and Genes.
(TIF)

Figure S2 **Pyrimidine metabolism in** ***Sinorhizobium meliloti*** **and ‘** ***Ca*** **. Liberibacter asiaticus’.** Enzymes annotated as present in both ‘*Ca*. Liberibacter asiaticus’ and *S. meliloti* are colored green. Enzymes annotated as present in *S. meliloti* but not in ‘*Ca*. Liberibacter asiaticus are colored orange. The metabolic pathway is from the Kyoto Encyclopedia of Genomes and Genes.
(TIF)

Table S1 **Frequency (%) of usage of amino acid codons in genomes of intracellular and free living members of the Rhizobiales.**
(RTF)

Table S2 **Amino acids over- and under-represented in the ‘** ***Ca*** **. Liberibacter asiaticus’ and** ***Bartonella henselae*** **proteomes as compared to the proteomes of free-living members of the** ***Rhizobiales*** **.**
(RTF)

Table S3 **Proteins involved in DNA repair pathways in ‘** ***Ca*** **. Liberibacter asiaticus and** ***Sinorhizobium meliloti*** **.**
(RTF)

Table S4 **Proteins that contain Ligase domains annotated on chromosomal and extra chromosomal elements of ‘** ***Ca*** **. Liberibacter asiaticus’ and other members of the Rhizobiales.**
(RTF)
