# Supplementary figures and images for: Comparison of the ‘Ca. Liberibacter asiaticus’ Genome Adapted for an Intracellular Lifestyle with Other Members of the Rhizobiales
Source: PLoS One. 2011 Aug 18;6(8):e23289. doi: 10.1371/journal.pone.0023289 (PMC3158068; doi:10.1371/journal.pone.0023289)

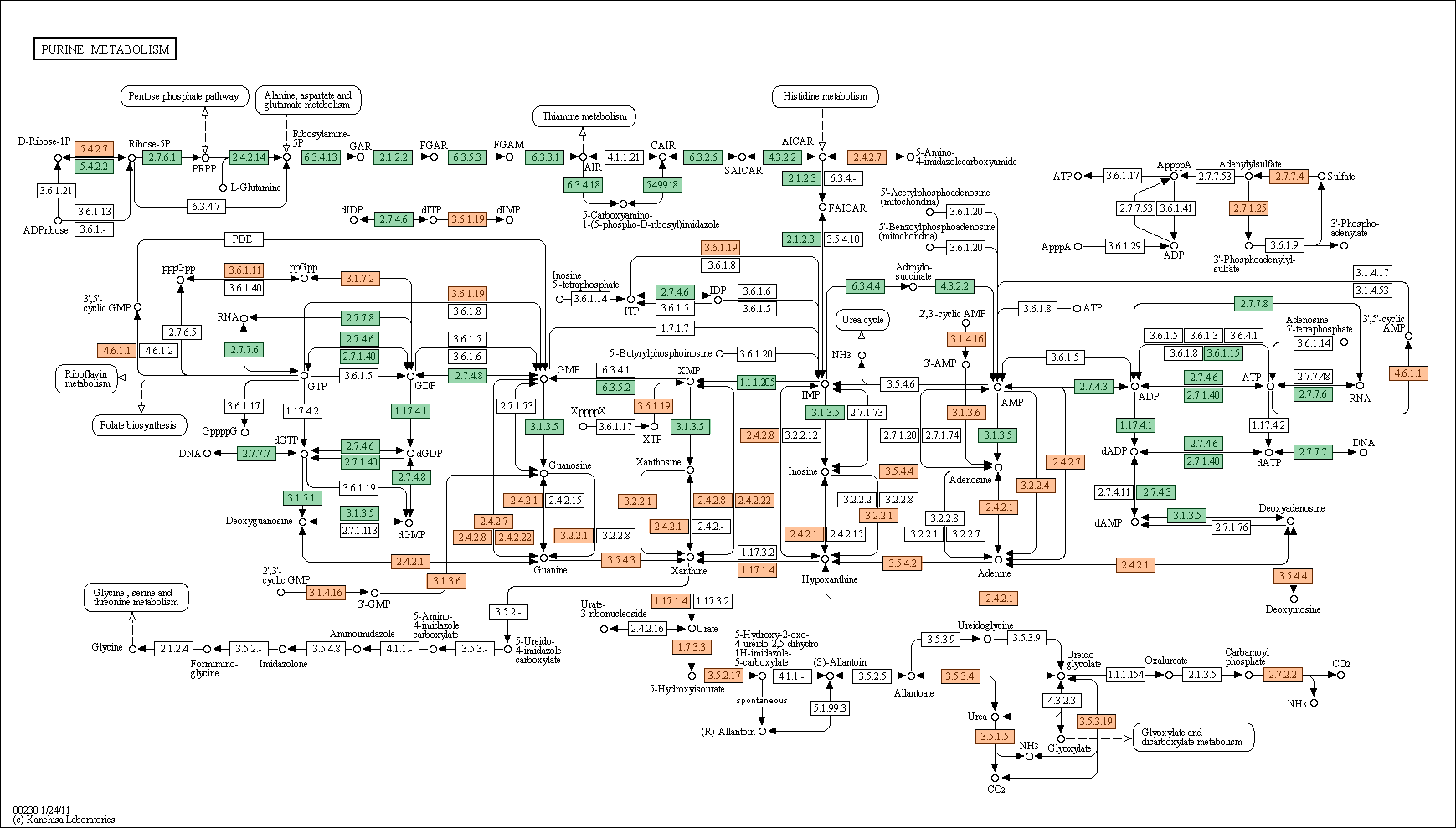

Supplement: Figure S1 — Purine metabolism in Sinorhizobium meliloti and ‘ Ca . Liberibacter asiaticus’. Enzymes annotated as present in both ‘Ca. Liberibacter asiaticus’ and S. meliloti are colored green. Enzymes annotated as present in S. meliloti but not in ‘Ca. Liberibacter asiaticus are colored orange. The metabolic pathway is from the Kyoto Encyclopedia of Genomes and Genes. (TIF) [file pone.0023289.s001.tif]

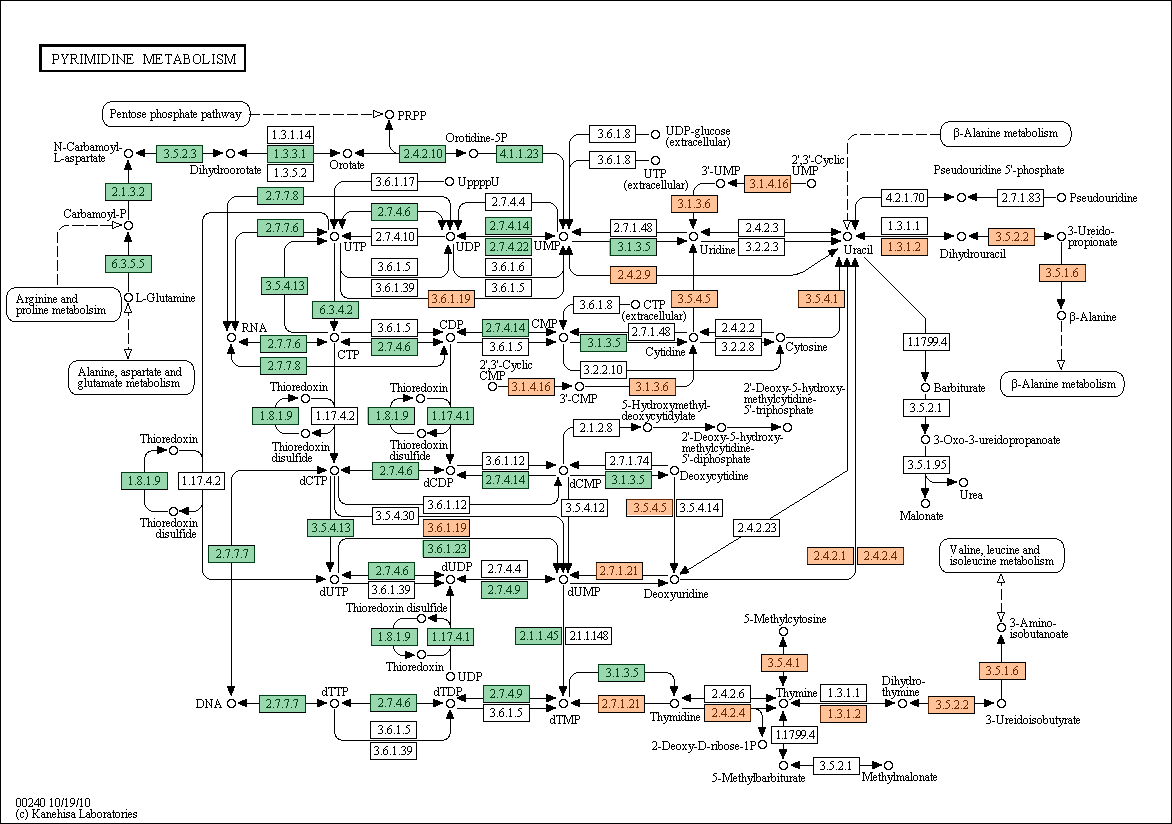

Supplement: Figure S2 — Pyrimidine metabolism in Sinorhizobium meliloti and ‘ Ca . Liberibacter asiaticus’. Enzymes annotated as present in both ‘Ca. Liberibacter asiaticus’ and S. meliloti are colored green. Enzymes annotated as present in S. meliloti but not in ‘Ca. Liberibacter asiaticus are colored orange. The metabolic pathway is from the Kyoto Encyclopedia of Genomes and Genes. (TIF) [file pone.0023289.s002.tif]
