# Supplementary material for: Comparison of the ‘Ca. Liberibacter asiaticus’ Genome Adapted for an Intracellular Lifestyle with Other Members of the Rhizobiales
Source: PLoS One. 2011 Aug 18;6(8):e23289. doi: 10.1371/journal.pone.0023289 (PMC3158068; doi:10.1371/journal.pone.0023289)
Supplement: Table S1 — Frequency (%) of usage of amino acid codons in genomes of intracellular and free living members of the Rhizobiales. (RTF) [file pone.0023289.s003.rtf]

Table S1.  Frequency (%) of usage of amino acid codons in genomes of intracellular and free living members of the Rhizobiales
Codon	Amino Acid	Liberibacter	Bartonella	Agrobacterium	Bradyrhizobium	Sinorhizobium	
_____________________________________________________________________________________________________________________	
TTT	Phe	79.15	83.47	26.67	17.55	16.06	
TTC	Phe	20.85	16.53	73.33	82.45	83.94	
TTA	Leu	31.52	26.38	0.83	0.72	0.36	
TTG	Leu	21.06	20.15	7.58	8.64	6.55	
CTT	Leu	23.49	30.99	17.41	7.94	13.75	
CTC	Leu	8.56	10.62	27.11	35.13	39.9	
CTA	Leu	9.61	5.47	1.42	1.63	1.1	
CTG	Leu	5.77	6.39	45.66	45.94	38.34	
TCT	Ser	40.85	31.38	5.46	3.47	3.59	
TCC	Ser	10	9.37	31.07	22.15	27.26	
TCA	Ser	17.33	21.95	5.37	4.91	3.24	
TCG	Ser	9.12	7.61	30.15	36.99	36.47	
AGT	Ser	15.69	18.15	3.8	3.8	3.4	
AGC	Ser	7.02	11.54	24.15	28.68	26.03	
TAT	Tyr	80.65	82.63	62.66	46.52	52.28	
TAC	Tyr	19.35	17.37	37.34	53.48	47.72	
TAA	Stop	51.13	52.78	26.15	16.11	16.76	
TAG	Stop	20.65	15.51	10.56	27.02	17.36	
TGA	Stop	28.22	31.72	63.29	56.87	65.88	
TGT	Cys	75.42	68.28	19.2	14.58	13.62	
TGC	Cys	24.58	31.72	80.8	85.42	86.38	
TGG	Trp	100	100	100	100	100	
CCT	Pro	48.66	40.62	10.07	7.11	7.35	
CCC	Pro	12.43	14.66	26.93	26.95	26.17	
CCA	Pro	29.2	33.55	6.32	6.44	4.1	
CCG	Pro	9.71	11.17	56.68	59.5	62.38	
CAT	His	79.1	77.69	56.7	44.66	48.22	
CAC	His	20.9	22.31	43.3	55.34	51.78	
CAA	Gln	72.96	72.22	15.28	18.03	14.69	
CAG	Gln	27.04	27.78	84.72	81.97	85.31	
CGT	Arg	36.14	43.19	18.27	9.25	10.56	
CGC	Arg	14	20.27	52.09	55.33	51.97	
CGA	Arg	14.19	10.34	3.56	5.84	4.55	
CGG	Arg	6.55	8.09	18.12	21.2	22.8	
AGA	Arg	21.09	12.92	2.84	2.33	2.87	
AGG	Arg	8.02	5.18	5.11	6.05	7.26	
ATT	lle	51.43	61.07	21.87	11.93	13.13	
ATC	lle	18.41	19.95	73.67	85.43	81.54	
ATA	lle	30.16	18.98	4.46	2.64	5.33	
ATG	Met	100	100	100	100	100	
ACT	Thr	34.08	23.99	4.81	4.59	3.98	
ACC	Thr	14.57	16.46	48.88	51.76	45.51	
ACA	Thr	35.13	43.84	8.64	5.97	5.76	
ACG	Thr	16.23	15.7	37.67	37.67	44.76	
AAT	Asn	79.81	76.67	43.29	31.11	34.26	
AAC	Asn	20.19	23.33	56.71	68.89	65.74	
AAA	Lys	72.58	75.02	27.92	16.32	18.61	
AAG	Lys	27.42	24.98	72.08	83.68	81.39	
GTT	Val	42.72	50.54	18.05	8.14	11.22	
GTC	Val	13.67	13.01	42.19	49.93	53.49	
GTA	Val	26.7	18.52	3.94	2.73	4.41	
GTG	Val	16.91	17.93	35.83	39.2	30.89	
GCT	Ala	39.88	37.61	9.36	6.16	6.84	
GCC	Ala	11.61	12.68	45.81	42.27	44.26	
GCA	Ala	33.74	36.48	11.86	8.75	11.02	
GCG	Ala	14.77	13.23	32.97	42.81	37.88	
GAT	Asp	86.05	85.16	49.78	33.51	34.75	
GAC	Asp	13.95	14.84	50.22	66.49	65.25	
GAA	Glu	74.3	75.31	58.21	29.84	42.14	
GAG	Glu	25.7	24.69	41.79	70.16	57.86	
GGT	Gly	32.35	39.3	21.36	10.01	12.07	
GGC	Gly	10.93	15.07	63.09	69.99	65.21	
GGA	Gly	40.59	30.29	6.6	8.52	9.73	
GGG	Gly	16.14	15.34	8.95	11.48	12.99	
