# Supplementary material for: Comparison of the ‘Ca. Liberibacter asiaticus’ Genome Adapted for an Intracellular Lifestyle with Other Members of the Rhizobiales
Source: PLoS One. 2011 Aug 18;6(8):e23289. doi: 10.1371/journal.pone.0023289 (PMC3158068; doi:10.1371/journal.pone.0023289)
Supplement: Table S2 — Amino acids over- and under-represented in the ‘ Ca . Liberibacter asiaticus’ and Bartonella henselae proteomes as compared to the proteomes of free-living members of the Rhizobiales . (RTF) [file pone.0023289.s004.rtf]

Table S2.  Amino acids over- and under-represented in the 'Ca. Liberibacter asiaticus' and Bartonella henselae proteomes as compared to the proteomes of free-living members of the Rhizobiales

			Occurrence in Proteome (%)		
		Liberibacter	Bartonella	Agrobacterium	Bradyrhizobium	Sinorhizobium	
Amino Acid							
UNDER REPRESENTED						
Proline		3.7	3.9	4.7	5.2	4.9	
Arginine		5.2	5.3	6.4	7.2	7.1	
Valine		6.4	6.3	7.3	7.5	7.4	
Glycine		6.2	6.8	8.3	8.3	8.5	
Alanine		6.3	8.1	11.5	12.4	12.1	
OVER REPRESENTED							
Phenylalanine		4.5	4.5	4.1	3.7	3.8	
Serine		8.1	6.6	5.8	5.8	5.5	
Glutamine		3.5	4	3.1	3.2	2.9	
Cysteine		1	0.8	0.1	0.1	0.1	
Isoleucine		8.8	7.4	5.8	5.2	5.5	
Asparagine		4.6	4.3	3	2.8	2.7	
Lysine		6.4	6.2	4.1	3.6	3.6	
