# Supplementary material for: Comparison of the ‘Ca. Liberibacter asiaticus’ Genome Adapted for an Intracellular Lifestyle with Other Members of the Rhizobiales
Source: PLoS One. 2011 Aug 18;6(8):e23289. doi: 10.1371/journal.pone.0023289 (PMC3158068; doi:10.1371/journal.pone.0023289)
Supplement: Table S3 — Proteins involved in DNA repair pathways in ‘ Ca . Liberibacter asiaticus and Sinorhizobium meliloti . (RTF) [file pone.0023289.s005.rtf]

Table S3. Proteins involved in DNA repair pathways in 'Ca. Liberibacter asiaticus' and Sinorhizobium meliloti.
ANNOTATION	Protein  in	Protein in 	E value to	L. asiaticus 	L. asiaticus	S. meliloti	S. meliloti	
	L. asiaticus	S. meliloti	L. asiaticus  to	pFAM 	E-value to	pFAM	E-value to	
			S. meliloti	Domains	pFAM model	Domains	pFAM model	
_____________________________________________________________________________________________________________________	
MISMATCH REPAIR								
mutS	ACT57223	CAC42832	0	MutS I	2.30E-68	MutS I	2.10E-73	
				MutS II	1.60E-21	MutS II	3.90E-35	
				MutSd	5.60E-70	MutSd	9.27E-78	
				MutSac	5.89E-112	MutSac	1.06E-113	
mutL	ACT56872	CAC45388	1.00E-174	HATPase C	6.20E-02	HATPase C	3.56E-01	
				DNA mis repair	7.90E-35	DNA mis repair	6.80E-34	
				MutL C	1.18E-48	MutL C	3.08E-52	
SSB	ACT56656	CAC46137	2.00E-46	SSB	3.60E-32	SSB	1.90E-31	
DNA Polä	ACT57277	CAC47912	2.00E-45	DNA Pol POL3ä	3.60E-04	DNA Pol POL3ä	3.30E-05	
Ligase	ACT57645	CAC46743	0	LIGANc	9.80E-172	LIGANc	1.30E-227	
	------------	CAC47322		N/A	N/A	DNA ligase AM	4.80E-31	
						DNA primase S	2.10E-24	
	-----------	CAC47487		N/A	N/A	DNA ligase A M	1.50E-18	
ExoDNAse VII	ACT57264	CAC41735	6.00E-132	tRNA anti	9.60E-09	tRNA anti	1.90E-13	
				Exonuc VII L	3.40E-79	Exo nuc VII L	2.50E-84	
recJ	ACT57374	CAC46300	6.00E-178	DHH	1.50E-46	DHH	5.10E-49	
				DHHA1	3.60E-11	DHHA1	2.10E-11	
NUCLEOTIDE EXCISSION							
uvrA	ACT56657	CAC46136	0	ABC tran	8.70E-20	ABC tran	1.40E-24	
uvrB	ACT57464	CAC46480	0	DEXDc	1.58E-24	DEXDc	6.89E-25	
				HELICc	9.53E-19	HELICc	3.56E-18	
				UVR	3.50E-12	UVR	2.60E-11	
uvrC	ACT57072	CAC45750	0	GIYc	2.14E-17	GIYc	3.72E-17	
				UvrC HhH N	5.90E-71	UvrC HhH N	5.80E-89	
uvrD	ACT57425	CAC46723	4.00E-157	UvrD helicase	5.10E-141	UvrD helicase	9.90E-180	
mfd	ACT57420	CAC46230	0	CarD TRCF	8.40E-39	CarD TRCF	1.40E-45	
				DEXDc	7.43E-30	DEXDc	8.54E-30	
				HELICc	5.78E-18	HELICc	3.93E-20	
				TRCF	4.00E-36	TRCF	1.40E-42	
DNA Pol I	ACT56750	CAC41562	0	53EXOc	7.69E-111	53EXOc	2.40E-121	
			HhH2		4.15E-11	HhH2	1.10E-15	
			POLAc		2.19E-106	POLAc	2.91E-120	
Ligase	ACT57645	CAC46743	0	LIGANc	9.80E-172	LIGANc	1.30E-227	
	-----------	CAC47322		N/A	N/A	DNA ligase AM	4.80E-31	
						DNA primase S	2.10E-24	
	-----------	CAC47487		N/A	N/A	DNA ligase A M	1.50E-18	
BASE EXCISSION								
ExoDNAse	ACT56761	CAC46098	1.00E-87	Exo endo phos	1.10E-44	Exo endo phos	3.30E-42	
	------------	CAC46228	N/A	N/A	N/A	Exo endo phos	1.20E-40	
	------------	CAC47780	N/A	N/A	N/A	Exo endo phos	2.40E-43	
DNA Pol I	ACT56750	CAC41562	0	53EXOc	7.69E-111	53EXOc	2.40E-121	
				HhH2	4.15E-11	HhH2	4.11E-14	
				POLAc	2.19E-106	POLAc	2.91E-120	
	ACT56701	CAC49694	8.00E-94	TerC	6.20E-06	TerC	4.70E-07	
				DUF475	1.10E-85	DUF475	8.80E-129	
Endonuc III	ACT56856	CAC41555	1.00E-79	ENDO3c	5.85E-52	ENDO3c	2.37E-61	
DNA Glycosyl	ACT57098	CAC41802	3.00E-82	Fapy DNA glyco	2.51E-34	DNA glycol	5.53E-54	
				H2TH	1.40E-19	H2TH	2.10E-18	
Ligase	ACT57645	CAC46743	0	LIGANc	9.80E-172	LIGANc	1.30E-227	
	-----------	CAC47322		N/A	N/A	DNA ligase AM	4.80E-31	
						DNA primase S	2.10E-24	
	-----------	CAC47487		N/A	N/A	DNA ligase A M	1.50E-18	
