# Supplementary material for: Comparison of the ‘Ca. Liberibacter asiaticus’ Genome Adapted for an Intracellular Lifestyle with Other Members of the Rhizobiales
Source: PLoS One. 2011 Aug 18;6(8):e23289. doi: 10.1371/journal.pone.0023289 (PMC3158068; doi:10.1371/journal.pone.0023289)
Supplement: Table S4 — Proteins that contain Ligase domains annotated on chromosomal and extra chromosomal elements of ‘ Ca . Liberibacter asiaticus’ and other members of the Rhizobiales. (RTF) [file pone.0023289.s006.rtf]

Table S4.  Proteins that contain Ligase domains annotated on chromosomal and extra chromosomal elements of 'Ca. Liberibacter asiaticus' and other members of the Rhizobiales.

Bacterium	Source	Annotation	pFam domain	Amino 	pFAM model	
and Protein				Acids	E-value	
						
_____________________________________________________________________________________________________________________	
						
Sinorhizobium						
CAC47322	Chromosome	Probable ATP-dependent DNA ligase	DNA_ligase_A_M	865	4.80E-31	
CAC47487	Chromosome	Putative DNA ligase	DNA_ligase_A_M	537	1.50E-18	
CAC46743	Chromosome	Probable DNA ligase	LIGANc	717	1.30E-227	
AAK64879	pSymA	conserved hypothetical protein	DNA_ligase_A_M	556	1.10E-27	
AAK64882	pSymA	ATP-dependent DNA ligase	DNA_ligase_A_M	346	7.00E-33	
AAK64883	pSymA	conserved hypothetical protein	Ku	268	3.20E-62	
CAC49792	pSymB	putative DNA ligase	DNA_ligase_A_M	818	6.50E-30	
CAC49016	pSymB	putative ATP-dependent DNA ligase	DNA_ligase_A_M	636	3.00E-26	
CAC49610	pSymB	putative ATP-dependent DNA ligase	DNA_ligase_A_M	364	1.30E-36	
CAC48411	pSymB	DNA ligase (ATP)	DNA_ligase_A_M	355	4.60E-23	
CAC49793	pSymB	putative DNA ligase	Ku	294	4.20E-67	
Agrobacterium 						
AAK86647	Chromosome	ATP-dependent DNA ligase	DNA_ligase_A_M	541	8.70E-22	
AAK87832	Chromosome	DNA ligase	LIGANc	724	1.69E-226	
AAK88815	Linear chromosome	ATP-dependent DNA ligase	DNA_ligase_A_M	771	9.60E-22	
AAK90430	At plasmid	ATP-dependent DNA ligase	DNA_ligase_A_M	884	6.10E-29	
AAK90473	At plasmid	ATP-dependent DNA ligase	DNA_ligase_A_M	350	1.60E-29	
AAK90426	At plasmid	ATP-dependent DNA ligase	DNA_ligase_A_M	345	1.60E-38	
AAK91050	Ti plasmid	ATP-dependent DNA ligase	DNA_ligase_A_M	353	6.90E-28	
Bradyrhizobium 						
BAC52038	Chromosome	probable DNA ligase	DNA_ligase_A_M	892	2.60E-24	
BAC53287	Chromosome	similar to DNA ligase	DNA_ligase_A_M	306	8.30E-31	
BAC53296	Chromosome	similar to DNA ligase	DNA_ligase_A_M	316	9.20E-42	
BAC46409	Chromosome	putative ATP dependent DNA ligase	DNA_ligase_A_M	724	7.30E-26	
BAC48385	Chromosome	putative ATP-dependent DNA ligase	Ku	327	3.10E-52	
BAC49882	Chromosome	similar to DNA ligase	DNA_ligase_A_M	180	4.90E-11	
BAC50447	Chromosome	similar to DNA ligase	DNA_ligase_A_M	210	1.50E-27	
BAC51856	Chromosome	DNA ligase	LIGANc	716	1.17E-220	
BAC52969	Chromosome	similar to DNA ligase	DNA_ligase_A_M	195	1.90E-18	
BAC50478	Chromosome	hypothetical protein	DNA_ligase_A_M	533	1.30E-10	
Rhizobium etli						
ACE89681	Chromosome	probable ATP-dependent DNA ligase	DNA_ligase_A_M	835	5.70E-28	
ACE90139	Chromosome	probable ATP-dependent DNA ligase	DNA_ligase_A_M	350	8.30E-35	
ACE90231	Chromosome	probable DNA ligase (ATP) protein	DNA_ligase_A_M	541	9.20E-29	
ACE91934	Chromosome	DNA ligase (NAD+) protein	LIGANc	718	3.04E-228	
Liberibacter						
ACT57645	Chromosome	NAD-dependent DNA ligase LigA	LIGANc	731	9.68E-172	
ACT56604	Chromosome	DNA ligase, NAD-dependent	No Domains	119	    NA	
ACT57669	Chromosome	DNA ligase, NAD-dependent	No Domains	119	    NA	
